# Identification and validation of a commercial cryopreservation medium for the practical preservation of *Dirofilaria immitis* microfilaria

**DOI:** 10.1186/s13071-020-04257-1

**Published:** 2020-07-29

**Authors:** Takahiro Shirozu, Akira Soga, Shinya Fukumoto

**Affiliations:** National Research Center for Protozoan Diseases, Obihiro University of Agriculture and Veterinary Medicine, Obihiro, Hokkaido 080-8555 Japan

**Keywords:** *Aedes aegypti*, Cryopreservation media, CultureSure, *Dirofilaria immitis*, Heartworm disease, Parasitic nematode, Microfilariae

## Abstract

**Background:**

*Dirofilaria immitis* is a parasitic nematode transmitted by mosquitoes and the cause of heartworm disease in dogs and dirofilariasis in humans and other mammals. The parasite is endemic worldwide. Vector stage research requires a reliable supply of *D. immitis* microfilariae (mf). It is believed that cryopreserved mf would retain viability and provide a powerful tool for vector stage research. However, reports on cryopreservation of *D. immitis* mf are limited. Therefore, this study aimed to validate commercial cryopreservation media to establish a practical, convenient and reproducible storage procedure for *D. immitis* mf.

**Methods:**

Six different commercially available cryopreservation media were compared with the traditional polyvinylpyrrolidone-dimethyl sulfoxide (PVP-DMSO) preservation solution. *In vitro* viability of purified *D. immitis* mf and mf-infected total blood was analyzed using a motility assay and propidium iodide staining. *In vivo* infectivity of *Aedes aegypti* mosquitoes with cryopreserved mf was assessed using a mosquito survival test and quantifying the number of third-stage larvae (L3) after 13 days post-infection.

**Results:**

Purified mf cryopreserved in CultureSure showed the best viability when compared to mf cryopreserved in the remaining five commercially available media and PVP-DMSO. Viability of mf in mf-infected total blood cryopreserved in CultureSure varied with the ratio of infected blood to CultureSure. Optimum results were obtained with 200 µl mf-infected blood:800 µl CultureSure. CultureSure was also the optimum medium for cryopreserving mf prior to infectivity of *A. aegypti.* The number of L3 was approximately the same for CultureSure cryopreserved mf (3× concentrated solution) and non-cryopreserved fresh mf.

**Conclusions:**

CultureSure is an optimal commercial cryopreservation solution for the storage of *D. immitis* purified mf, mf-infected total blood, and mf used for *in vivo* mosquito experiments. Furthermore, this study describes an easy preservation method for clinical *D. immitis*-infected blood samples facilitating vector stage studies, as well as the study of macrocyclic lactone resistance in heartworms and the education of veterinarians. 
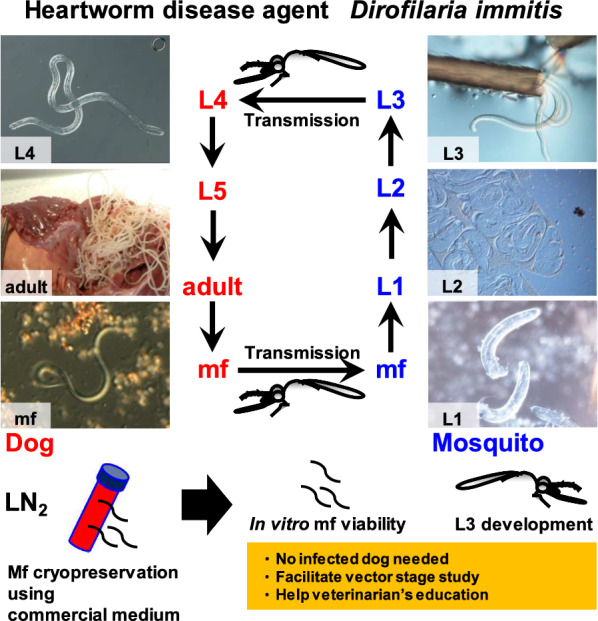

## Background

The parasitic nematode *Dirofilaria immitis* is transmitted by mosquitoes causing potentially lethal heartworm disease in dogs, pulmonary dirofilariasis in humans, and dirofilariasis in various mammals [1]. Wild carnivores are also sentinels for the spread of *D. immitis*. Despite the availability of effective preventive medicines, *D. immitis* is endemic worldwide. Therefore, a better understanding of host-parasite interactions between mosquito vectors and *D. immitis* is needed to establish a truly effective control strategy.

Vector stage experiments require live *D. immitis* microfilariae (mf) obtained from infected animals [2]. Having a reliable supply of live mf on demand is very important. Cryopreservation is a powerful tool allowing pathogens to be stored in a viable state. However, there are only a few reports describing cryopreservation of *D. immitis* mf. Initially, in 1947, Weinman and McAllister [3] demonstrated cryopreservation of mf in citrated or heparinized blood without cryoprotectant additives. Taylor [4] in 1960 and Lok et al. [5] in 1983 used glycerol and dimethylsulfoxide (DMSO), respectively, for cryopreservation of mf and assessed the subsequent infectivity of mosquitoes. In the most recent study, published in 2001, Bartholomay et al. [6] prepared and tested a 16% polyvinylpyrrolidone (PVP)-6% DMSO solution (PVP-DMSO) as a cryoprotective medium.

In the previous studies, the cryopreservation media tested were originally prepared by the investigators. Recently, various commercial cryopreservatives have become available and they are commonly used, especially for mammalian cell cryopreservation. For convenience, reproducibility and standardization purposes, we sought to identify a commercially available cryopreservation medium for effective *D. immitis* mf preservation. To this end, here, we compared six commercial cryopreservation media with the traditional PVP-DMSO preservative [6] for the cryopreservation of *D. immitis* mf. Effectiveness was evaluated by measuring *in vitro* viability and *in vivo* infectivity of mosquitoes.

## Methods

### Animals

Female beagles (2–4 years-old) were experimentally infected with *D. immitis* SF1 strain originally isolated in Japan [7] and reared under a veterinarian’s administration. *Aedes aegypti* Liverpool (LVP)-OB strain, kindly provided by Dr R Maeda, the Obihiro University of Agriculture and Veterinary Medicine (OUAVM), Japan, susceptible to *D. immitis* infection was maintained at 27 ℃ and > 80% humidity on a diet of 5% fructose and a 12:12 h light:dark photoperiod.

### Microfilariae purification and cryopreservation

Cryopreservation was initially tested using purified *D. immitis* mf isolated from heparinized blood of an experimentally infected dog. The mf were purified using a PD-10 gel purification column (GE Healthcare, Amersham Place, UK) equilibrated with RPMI 1640 Medium (Gibco 2120429, Thermo Fisher Scientific, Waltham, MA, USA) supplemented with 1× penicillin-streptomycin (Gibco 15140122) [8, 9]. Purified mf were centrifuged at 1000×*g* for 10 min at room temperature. The supernatant was discarded and the pelleted mf were re-suspended in 1 ml of cryopreservation medium (*c.*3000 or 15,000 mf/tube) and transferred to cryogenic tubes (Nunc 119546, Thermo Fisher Scientific). Samples were stored in freezing containers (Corning® CoolCell® LX, Corning, NY, USA) overnight at − 80 ℃ before being transferred into liquid nitrogen, and stored until analysis. Cryopreservation media used in this study were as follows: PVP-DMSO (0.004 M PVP, average molecular weight of 40,000 and 6% DMSO) in phosphate buffered saline (PBS) [6, 10], CultureSure® Freezing Medium (CultureSure, FUJIFILM Wako, Osaka, Japan), Cellbanker® 1 (Cellbanker, Zenoaq Resource, Shiga, Japan), LaboBanker 1 (LaboBanker, Kurabo, Osaka, Japan), Cos banker (Cosmo Bio, Tokyo, Japan), Bambanker® (GC Lymphotec, Tokyo, Japan), CryoScarless® DMSO-Free (CryoScarless, Bio Verde, Kyoto, Japan), and RPMI 1640 (Table 1).

**Table 1:** Information about the composition of commercial cryopreservation media

Product name	DMSO (%)	Bovine serum	Manufacturer
Cellbanker 1	10	< 80%	Zenoaq Resource
LaboBanker 1	10	Containing^a^	Kurabo
CultureSure Freezing Medium	10	Free (containing BSA)	Fujifilm
Bambanker	10	Free (containing bovine serum-derived components)	GC Lymphotec
Cos banker	Containing^a^	Free	Cosmo Bio
CryoScarless DMSO-Free	Free	Free	Bio Verde

^a^The concentration is not stated by the manufacturers

### *In vitro* motility assay

Cryopreserved mf were thawed rapidly by swirling in a water bath at 37 ℃. Immediately after thawing, the mf were suspended in 10 ml of RPMI 1640 medium and centrifuged at 1500×*g* for 10 min at room temperature. The pelleted mf were re-suspended in 1 ml of RPMI 1640 medium containing 5% fetal calf serum, transferred to 12-well cell culture plates (Nunc) and incubated in 5% CO_2_ in air at 37 ℃ for 3 days. The motility of mf was assessed microscopically on days 0, 1 and 3 post-thawing (dpt) (*n* ≥ 3) and subjectively scored as inactive or dead (−), less active (+), moderately active (++) and highly active (+++) [11]. The results were statistically analyzed to rank the various media using a Chi-square test and adjusted residuals (AR). Absolute AR values were considered significant at AR ≥ 1.96. The difference between PVP-DMSO and each cryopreservation medium was analyzed using Welch’s t-test.

### Cryopreservation of *D. immitis* mf-infected total blood

Fresh, heparinized blood (50–400 µl) was collected from *D. immitis*-infected dogs (*c.*25,000 mf/ml) and mixed with CultureSure up to 1 ml and then cryopreserved as described above. Differences in motility between purified mf and mf-infected total blood were analyzed by one-way analysis of variance (ANOVA), followed by Tukey’s multiple comparisons test.

### Propidium iodide (PI) staining assay

To evaluate any physical damage caused by cryopreservation, cultured mf were stained with PI (2 µg/ml) (Molecular probes, Eugene, OR, USA) for 10 min on 3 dpt. The stained mf were washed with PBS by centrifugation at 1500×*g* for 10 min at 4 ℃ and then subjected to fluorescence microscopy observation using the same exposure time in each experiment. Methanol-fixed mf were used as a positive control. The results were statistically compared using a Chi-square test and calculated AR.

### *In vivo* third-stage larva (L3) development assay

Mf cryopreserved with commercial CultureSure, LaboBanker media and traditional PVP-DMSO were used to determine the infectivity of mosquitoes *in vivo*. Thawed mf were fed to female *A*. *aegypti* on day 7 post-emergence for 2 h by artificial membrane feeding using Parafilm (Bemis company, Neenah, WI, USA). Blood-feeding experiments were completed within 4 h post-thawing. The concentration of thawed mf was adjusted to 18 mf/µl using heparinized blood collected from a healthy dog. Non-cryopreserved fresh blood (6 mf/µl) was collected from an infected dog and used as the control. Fully fed mosquitoes were selected under CO_2_ anesthesia and maintained for 13 d. Mosquito survival was monitored every day. At day 13 post-infection (dpi), all surviving female mosquitoes were dissected under a stereomicroscope to investigate the number of L3. Mosquito survival was analyzed by Chi-square test and the L3 count was analyzed by Welch’s t-test.

### Statistical analyses

All statistical analyses were performed using GraphPad Prism 7 (GraphPad, San Diego, CA, USA). A significant difference was defined by *P *< 0.05 and *P *< 0.10 was considered a marginal difference.

## Results and discussion

### *In vitro* viability of purified microfilariae

**Fig. 1:**
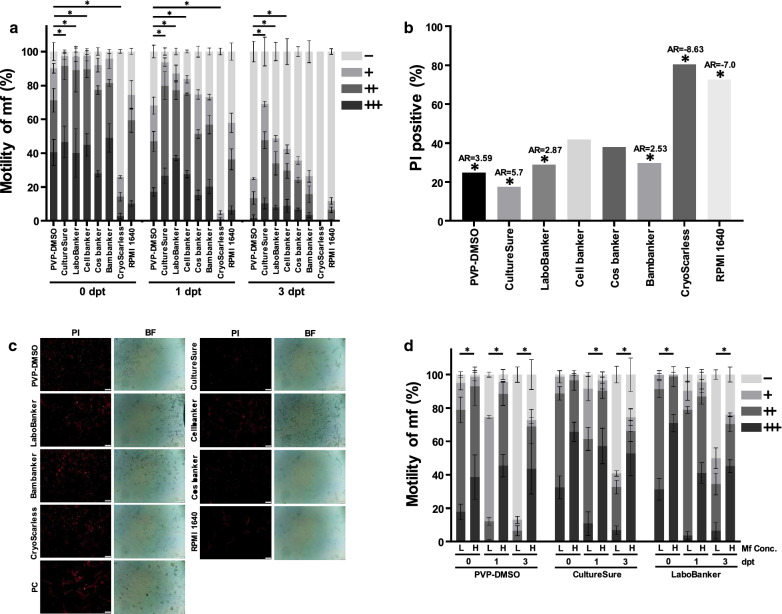
Validation of commercial cryopreservation media for the preservation of *D. immitis* microfilariae (mf) *in vitro*. **a** Comparison of mf motility *in vitro* for various cryopreservation media. Motility of mf (*c.*3000 mf/ml) was analyzed on days 0, 1 and 3 post-thawing (dpt) and scored at four levels as follows: inactive or dead (-), less active (+), moderately active (++) and highly active (+++). An asterisk indicates a significant difference (*P* < 0.05) using Welch’s t-test between the PVP-DMSO control and the other cryopreservation media. Error bars indicate ± standard deviation (SD). **b** Percent of mf showing positive propidium iodide (PI) staining 3 dpt, indicating dead or dying mf. An asterisk indicates a significant difference based on Chi-square test and adjusted residuals (AR). An absolute value of AR ≥ 1.96 was considered significant (*P* < 0.05). **c** Fluorescent images of PI staining of mf 3 dpt (exposure, 200 ms; gain, 0.5). PI image (left) and bright field (BF) image (right). PC, positive control for PI staining fixed by methanol. *Scale-bars*: 100 µm. **d** Comparison of mf motility at high (H; *c.*15,000 mf/ml) and low (L; 3000 mf/ml) concentrations during cryopreservation. Motility was scored at four levels as described in panel a. An asterisk indicates a significant difference (*P* < 0.05) by Welch’s t-test. Error bars indicate ± SD

The viability of cryopreserved mf was compared between traditional PVP-DMSO and several commercially available cryopreservation media. Microscopic observation revealed that mf cryopreserved with CultureSure exhibited the highest motility (*χ*^2^ = 721.2, *df* = 7, *P* < 0.0001, AR = 8.5 on 0 dpt; *χ*^2^ = 648.5, *df* = 7, *P* < 0.0001, AR = 10.51 on 1 dpt and *χ*^2^ = 333.3, *df* = 7, *P* < 0.0001, AR = 12.19 on 3 dpt) (Fig. 1a). Mf cryopreserved with LaboBanker exhibited the second highest motility (AR = 7.67 on 0 dpt, 9.88 on 1 dpt, and 6.55 on 3 dpt), and Mf in CryoScarless exhibited the lowest motility (AR = − 24.03 on 0 dpt, − 20.06 on 1 dpt, and − 10.12 on 3 dpt). When mf motility was compared based on moderately active and highly active levels, mf in CultureSure (*t* = 6.634, *df* = 2.214, *P* = 0.017 on 0 dpt; *t* = 6.682, *df* = 3.118, *P* = 0.0061 on 1 dpt and *t* = 6.76, *df* = 3.835, *P* = 0.0029 on 3 dpt), LaboBanker (*t* = 4.495, *df* = 3.934, *P* = 0.011 on 0 dpt; *t* = 5.944, *df* = 3.373, *P* = 0.0068 on 1 dpt and *t* = 4.493, *df* = 3.997, *P* = 0.011 on 3 dpt) and Cellbanker (*t* = 5.024, *df* = 3.571, *P* = 0.0099 on 0 dpt; *t* = 6.184, *df* = 2.383, *P* = 0.016 on 1 dpt and *t* = 3.02, *df* = 3.698, *P* = 0.043 on 3 dpt) media all exhibited significantly higher motility on 0, 1 and 3 dpt than mf preserved in traditional PVP-DMSO media. Mf in CryoScarless medium showed significantly lower motility compared to PVP-DMSO on 0 (*t* = 18.87, *df* = 2.116, *P* = 0.0022) and 1 dpt (*t* = 10.61, *df* = 2.071, *P* = 0.0078).

The viability of mf on 3 dpt was quantified based on the ability of PI to penetrate dead or dying cells but not living cells. CultureSure-cryopreserved mf showed significantly lower PI values compared to all of the other cryopreservation media (*χ*^2^ = 161.3, *df* = 7, *P* < 0.0001, AR = 5.7) (Fig. 1b). The mf cryopreserved in PVP-DMSO (AR = 3.59), LaboBanker (AR = 2.87) and Bambanker (AR = 2.53) showed significantly lower PI positive rates (Fig. 1b). These results are supported by fluorescent imaging of mf PI staining in each medium (Fig. 1c). Translating our results, to do a parallel with other published studies, the total motile parasite ratio of CultureSure-cryopreserved mf after thawing (97.2%, Fig. 1a) was considered equivalent or slightly better than the reported for cryopreserved mf using 5% DMSO medium (78.8–91.6%) [5] or PVP-DMSO (> 95%) [6]. From these experiments, it is clear that CultureSure is the optimal cryopreservation medium tested for the preservation of *D. immitis* mf.

Parasite concentration levels were a factor influencing motility in the various media. In general, motility of mf cryopreserved at a high concentration (*c.*15,000 mf/ml) was significantly greater than mf cryopreserved at a low concentration (*c.*3000 mf/ml). This was true for mf cryopreserved in PVP-DMSO on 0 to 3 dpt (*t* = 4.451, *df* = 3.499, *P* = 0.015 on 0 dpt; *t* = 36.17, *df* = 2.929, *P* < 0.0001 on 1 dpt and *t* = 11, *df* = 2.414, *P* = 0.004 on 3 dpt), in CultureSure on 1 (*t* = 4.708, *df* = 3.817, *P* = 0.010) and 3 dpt (*t* = 3.805, *df* = 2.865, *P* = 0.035) and in LaboBanker on 0 (*t* = 4.748, *df* = 3.291, *P* = 0.014) and 3 dpt (*t* = 6.522, *df* = 2.901, *P* = 0.008) (Fig. 1d). These results suggest that cryopreservation of mf should be carried out at the higher mf concentration to retain better viability.

### *In vitro* viability of microfilariae in mf-infected total blood

**Fig. 2:**
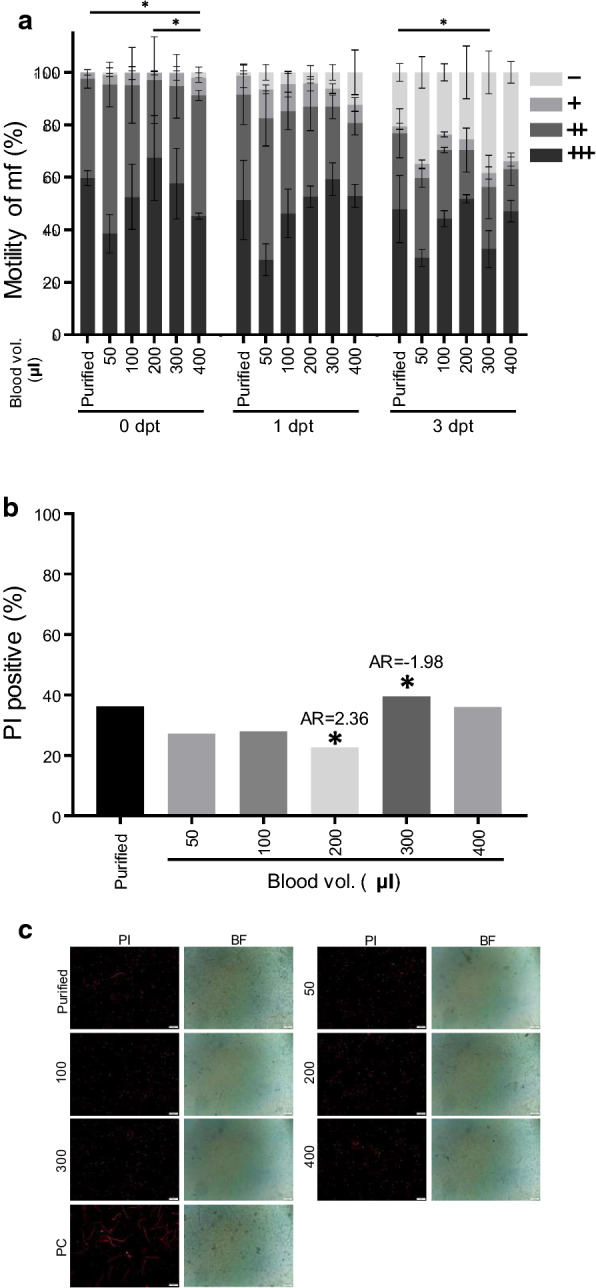
Viability of cryopreserved *D. immitis* microfilariae (mf)-infected total blood. **a***In vitro* motility was scored over a range of blood volumes (X-axis) in CultureSure (1 ml total volume) and compared to purified mf at 0, 1 and 3 days post-thawing (dpt). Scoring was at four levels as follows: inactive or dead (-), less active (+), moderately active (++) and highly active (+++). An asterisk indicates a significant difference (*P* < 0.05) by one-way ANOVA, followed by Tukey’s multiple comparisons test. Error bars indicate ± SD. **b** Percent positive PI staining of mf-infected total blood on 3 dpt. An asterisk indicates a significant difference by Chi-square test and adjusted residual (AR). AR ≥ 1.96 was considered significant (*P* < 0.05). **c** Fluorescent images of propidium iodide (PI) staining of mf-infected total blood on 3 dpt (exposure, 100 ms; gain, 0.5). PI image (left) and bright field (BF) image (right). *Abbreviation*: PC, positive control. *Scale-bars*: 100 µm

Cryopreservation of *D. immitis* mf-infected total blood is especially important in clinical settings. The motility of mf in mf-infected total blood was compared over a range of blood volumes in CultureSure (1 ml total volume) and with purified mf (*c.*25,000 mf/ml) (Fig. 2a). The results were comparable to purified mf cryopreservation, in most cases. Significant differences were observed between purified mf and mf-infected total blood volumes of 400 µl on 0 dpt (*q* = 5.431, *df* = 12, *P* = 0.022) and 300 µl on 3 dpt (*q* = 5.38, *df* = 12, *P* = 0.024). In addition, a significant difference was seen for mf-infected volumes of 200 µl and 400 µl on 0 dpt (*q* = 5.048, *df* = 12, *P* = 0.035). Analysis of PI staining of mf on 3 dpt, demonstrated that mf-infected total blood at a volume of 200 µl showed significantly lower PI positive values (*χ*^2^ = 12.19, *df* = 5, *P* = 0.032, AR = 2.36, Fig. 2b), whereas the 300 µl sample showed the highest PI staining values (AR = 1.98, Fig. 2b). These findings were supported by fluorescent imaging (Fig. 2c). Altogether, these results suggest that cryopreservation is optimal with 200 µl (*c.*25 mf/µl) of infected blood brought to a total volume of 1 ml with CultureSure.

### Vector infectivity and survival of cryopreserved microfilariae

**Fig. 3:**
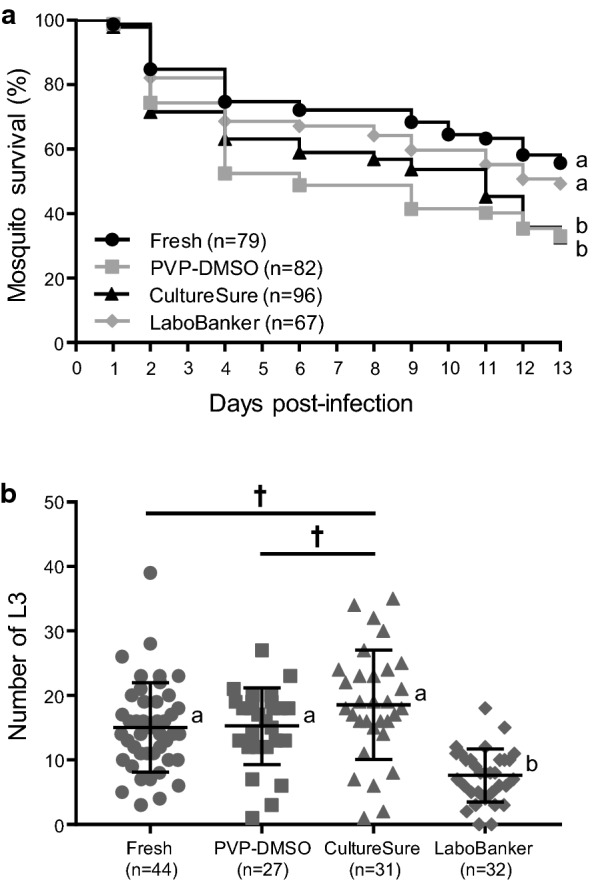
Analysis of *in vivo* infectivity of *A*. *aegypti* mosquitoes with cryopreserved *D. immitis* microfilariae (mf). **a** Survival of mosquitoes infected with mf cryopreserved in PVP-DMSO, CultureSure and LaboBanker and non-cryopreserved control (Fresh) over 13 d post-infection (dpi). The survival rate on 13 dpi was statistically compared using a Chi-square test. Different letters (a, b) indicate significant differences (*P* < 0.05). **b** The number of third-stage larvae (L3) in the mosquitoes on 13 dpi. Each plot shows the L3 of individual mosquitoes. Error bars = mean ± SD. The L3 were statistically compared using Welch’s t-test. Significant differences (*P* < 0.05) were observed between different letters (a, b); † indicate marginal differences

Infectivity of mosquito vectors with *D. immitis* mf cryopreserved with CultureSure or LaboBanker was compared with traditional PVP-DMSO cryopreservation. Non-cryopreserved, fresh mf were used as a positive control. The survival rate of mosquitoes fed LaboBanker cryopreserved mf was significantly higher compared to mosquitoes fed CultureSure (*χ*^2^ = 4.761, *df* = 1, *P* = 0.029) or PVP-DMSO (*χ*^2^ = 4.086, *df* = 1, *P* = 0.043) cryopreserved mf on 13 dpi (Fig. 3a). The number of L3 in mosquitoes fed CultureSure cryopreserved mf tended to be higher than that of the L3 for mf cryopreserved in PVP-DMSO (*t* = 1.728, *df* = 53.64, *P* = 0.090) and fresh (*t* = 1.897, *df* = 56.14, *P* = 0.063) mf (Fig. 3b). Although different mosquito colonies and mf infectious inocula were used, the mf infectivity reported here was similar to the one previously reported for PVP-DMSO cryopreserved mf [6]. The ratios of mean L3 numbers in individual mosquitoes/feeding inoculum concentration were 0.85 and 0.77 for PVP-DMSO cryopreserved mf in our study and a previous study, respectively [6]. Remarkably, this ratio, determined for CultureSure was 1.03. The number of L3 of mosquitoes fed LaboBanker cryopreserved mf was significantly lower compared to the other treatments (Fresh: *t* = 5.868, *df* = 71.41, *P* < 0.0001; PVP-DMSO: *t* = 5.67, *df* = 45, *P* < 0.0001; and CultureSure: *t* = 6.496, *df* = 42.96, *P* < 0.0001). These results indicate that CultureSure is an optimal medium for *D. immitis* mf cryopreservation. Lok et al. [5] demonstrated that 6.0–20.0% of mf cryopreserved in 5% DMSO medium reached the L3 stage when inoculated to mosquitoes using the enema method. The estimated average blood volume ingested by *A. aegypti* per blood meal was 2.3–2.6 µl [12, 13]. In our study, assuming a blood intake of 2.5 µl (18 mf/µl) per mosquito, the L3 development ratio was calculated as 41.8% (18.8 L3/45 mf), suggesting CultureSure is better than 5% DMSO medium for the cryopreservation of *D. immits* mf.

### Importance of DMSO in cryopreservation media

DMSO appears to be an important component in cryopreservation media for *D. immitis* mf. In the present study, most of the commercial cryopreservation media examined yielded a positive performance, except for CryoScarless, which was the only medium that did not contain DMSO. DMSO is known to inhibit ice crystal formation during the freezing process and it is commonly used for mammalian cell cryopreservation [14]. Selection of a DMSO-containing medium seems critical for successful cryopreservation of *D. immitis* mf.

### Justification and practical applications for using cryopreserved microfilariae

Preparation of *D. immitis*-infected animals and fresh mf is a bottleneck for *in vivo* mosquito vector experiments. Here, we demonstrated that cryopreserved mf could be used for *in vivo* mosquito experiments instead of fresh mf, thereby eliminating the need to maintain infected animals. The concentration of cryopreserved mf fed to mosquitoes in mf-infected blood was adjusted to 3-fold higher levels than non-cryopreserved, fresh mf. Under these conditions, the number of L3 for the mosquitoes was almost equivalent to that of fresh mf.

Standardization of *D. immitis* mf cryopreservation method is essential for the study of drug-resistant *D. immitis*. Macrocyclic lactones have been widely used to prevent heartworm infections for the past three decades [15]. Since the emergence of macrocyclic lactone resistance in *D. immitis* in 2005 [16], numerous field and laboratory-induced isolates have been studied [17–19]. These isolates are essential for further studies to completely understand drug resistance in *D. immitis.* However, it is quite expensive and time-consuming to maintain each of these isolates by continuous alternate passages in mosquitoes and dogs. Although cryopreservation methods are not without limitations, such as the need for continuous and relatively frequent replenishment of liquid nitrogen levels, they offer unique advantages over continuous biological maintenance. In addition and importantly, cryopreservation allows the reduction of animal use (fewer dogs are needed), respecting the ethics in animal experimentation, and makes the continuous maintenance of an insectary for mosquito production expendable.

Cryopreservation of *D. immitis* mf has practical applications for veterinary science education [20]. Currently, diagnosis of *D. immitis* infection is usually performed with a commercial antigen detection kit; however, false negatives are common [21]. Adequate skills need to be developed to ensure mf detection in clinical settings. The supply of *D. immitis* mf available for veterinary education curricula is limited due to the difficulty of maintaining live animals infected with *D. immitis* mf. We believe our results validate the use of a commercial cryopreservation medium for *D. immitis* mf, overcoming the current issues.

## Conclusions

In summary, we determined that CultureSure is an optimal commercial cryopreservation solution for the storage of *D. immitis* mf. CultureSure was successful in cryopreserving purified mf, mf-infected total blood, and mf for *in vivo* mosquito vector experiments. This study will facilitate not only the study of the vector stage of *D. immitis*, but also the study of macrocyclic lactone resistance in heartworms and the consequent education of veterinarians.

## Data Availability

Data supporting the conclusions of this article are provided within the article.

## Abbreviations

ANOVA: analysis of variance
AR: adjusted residual
DMSO: dimethyl sulfoxide
dpi: days post-infection
dpt: days post-thawing
L3: third-stage larvae
LVP: Liverpool
mf: microfilariae
OUAVM: Obihiro University of Agriculture and Veterinary Medicine
PBS: phosphate buffered saline
PI: propidium iodide
PVP: polyvinylpyrrolidone
